# Structural constraints in the packaging of bluetongue virus genomic segments

**DOI:** 10.1099/vir.0.066647-0

**Published:** 2014-10

**Authors:** Christiane Burkhardt, Po-Yu Sung, Cristina C. Celma, Polly Roy

**Affiliations:** Department of Pathogen Molecular Biology, Faculty of Infectious and Tropical Diseases, London School of Hygiene and Tropical Medicine, London, UK

## Abstract

The mechanism used by bluetongue virus (BTV) to ensure the sorting and packaging of its 10 genomic segments is still poorly understood. In this study, we investigated the packaging constraints for two BTV genomic segments from two different serotypes. Segment 4 (S4) of BTV serotype 9 was mutated sequentially and packaging of mutant ssRNAs was investigated by two newly developed RNA packaging assay systems, one *in vivo* and the other *in vitro*. Modelling of the mutated ssRNA followed by biochemical data analysis suggested that a conformational motif formed by interaction of the 5′ and 3′ ends of the molecule was necessary and sufficient for packaging. A similar structural signal was also identified in S8 of BTV serotype 1. Furthermore, the same conformational analysis of secondary structures for positive-sense ssRNAs was used to generate a chimeric segment that maintained the putative packaging motif but contained unrelated internal sequences. This chimeric segment was packaged successfully, confirming that the motif identified directs the correct packaging of the segment.

## Introduction

*Bluetongue virus* (BTV) is the prototype species of the genus *Orbivirus* within the *Reoviridae*, a family consisting of non-enveloped viruses with segmented dsRNA genomes. The BTV genome consists of 10 dsRNA molecules designated S1–S10 (Verwoerd *et al.*, 1970) which are, together with the viral subcore proteins VP1 (RNA-dependent RNA polymerase), VP4 (RNA-capping enzyme) and VP6 (RNA helicase), enclosed in a double-layered protein shell, with an inner layer of core protein VP3 and an outer layer of VP7. An outer capsid composed of two proteins, VP2 and VP5, encapsidates the inner core. In addition to these seven structural proteins, BTV also synthesizes four non-structural proteins (NS1–NS4) in infected cells, each of which is involved in various stages of virus replication and assembly. In particular, NS2, which is the major component of viral inclusion bodies (VIBs), the site of the core assembly, possesses strong binding affinity for ssRNA secondary structures (Lymperopoulos *et al.*, 2006; Roy, 2008; Verwoerd *et al.*, 1972).

Upon attachment to the cell surface receptor, BTV is endocytosed, where the outer protein layer is removed and cores are released into the cytoplasm (Forzan *et al.*, 2004, 2007; Huismans *et al.*, 1987). The released cores then become transcriptionally active (Huismans *et al.*, 1987), releasing 10 positive-sense ssRNAs through the pores located at the 12 vertices of the icosahedral core (Diprose *et al.*, 2001). These ssRNA molecules act as templates (mRNAs) for synthesis of viral proteins as well as for genomic dsRNA segments for progeny virions. Assembly of nascent BTV cores takes place within VIBs in the cytoplasm (Eaton *et al.*, 1990).

Recently, using a cell-free assembly (CFA) *in vitro* assay, we have shown that assembly of BTV particles is initiated by the interaction of the three enzymic proteins (VP1, VP4 and VP6) with BTV ssRNA molecules. The protein–RNA complex is then coated with VP3 (termed the ‘subcore’), which subsequently interacts with VP7 to form the core, initiating the dsRNA synthesis and protecting the RNA from degradation (Lourenco & Roy, 2011).

The sequence of events in the morphogenesis of BTV makes it clear that sorting and packaging of viral RNA occurs at the ssRNA level. In order to ensure that every progeny virion contains the right set of 10 genomic segments, sorting and packaging of ssRNA molecules must be highly specific and regulated. The 10 genomic BTV segments possess variable numbers of nucleotides ranging from 822 bp for S10 up to 3944 bp for S1 (Fukusho *et al.*, 1989; Roy *et al.*, 1985). However, their length is highly conserved among different BTV serotypes (26 serotypes). Each segment has a 5′ and 3′ untranslated region (UTR) flanking the ORF of the protein(s) that each encodes. The 5′ UTR consists of a conserved pentanucleotide GUUAA and a non-conserved region of variable length ranging from 3 (S4) to 34 nt (S5). The 3′ UTR also consists of a conserved pentanucleotide CUUAC at the 3′ end and a region of variable length ranging from 24 (S1) to 113 nt (S10). Whilst some segments share the same 5′ UTR length, the lengths of the 3′ UTR are unique. Nevertheless, the exact length of the 3′ UTR of BTV segments is conserved between different serotypes, suggesting a strong constraint on this non-coding region. Interestingly, S6 (encoding VP5) and S2 (encoding VP2), which are the most variable segments in BTV and determinants of serotype specificity, differ in their 3′ UTR length by 1 and up to 3 nt, respectively.

Here, we undertook a study to elucidate the nature of the packaging signals of BTV ssRNAs, focusing on two segments, S4 of BTV-9 and S8 of BTV-1. Various assays were employed to investigate packaging of genome segments independently from other processes in the biology of the virus, such as infection, transcription or potential host-dependent influences. We determined the nature and the sequence requirements of the signals responsible for packaging. We also predicted and verified the minimal sequence requirement using a chimeric segment containing the ORF of an alternate segment, S8, in place of the ORF of S4. The results suggested a similar structural requirement in both segments.

## Results

### Mapping of the sequences on BTV ssRNAs essential for packaging

**Fig. 1.**
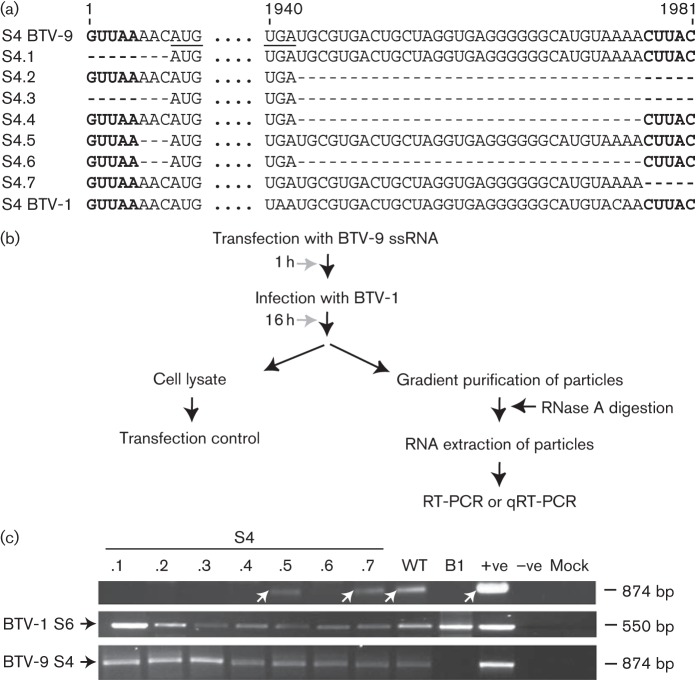
Mutations in S4 of BTV-9 5′ and 3′ UTRs tested by *in vivo* reverse transcription packaging assay. (a) Nucleotide sequences of ssRNA of mutant S4. Start and stop codons of VP4 ORF are underlined; dashes indicate nucleotide deletions. Deletion mutants S4.1–S4.7 are indicated. (b) A cartoon showing the reverse transcription packaging assay. RT-PCR, reverse transcription PCR; qRT-PCR, quantitative real-time PCR. (c) Detection of mutant S4 in the reverse transcription packaging assay. Upper panel: packaged RNA was purified and S4 was detected by reverse transcription PCR yielding an 874 bp product (white arrows) for mutants S4.1–S4.7 or WT S4. RNA from cells infected with BTV-1 but not transfected (B1). +ve, S4 of BTV-9; –ve, no RNA; mock, untreated cells. Middle panel: as internal control, S6 of BTV-1 was detected by reverse transcription PCR (550 bp product). +ve, S6 RNA. Lower panel: as transfection control, S4 of BTV-9 was similarly detected from transfected/infected cell lysate. For consistency, each experiment was performed three times.

As the length of the 3′ UTR differs between the 10 segments of BTV, but is highly conserved among the same segments of different serotypes, it was hypothesized that the packaging signals could be located in the 3′ UTR. To test this hypothesis, we initially targeted one ssRNA segment (S4) of BTV-9 as a representative of BTV ssRNAs and used an RNA packaging assay that allowed packaging of a single ssRNA by an infectious virus (Fig. 1b). A series of mutations on S4 ssRNA was introduced using *in vitro* T7 transcription of the S4 cDNA clone and mutant ssRNAs were tested for their capacity to be packaged into viral particles. To this end, BSR cells were first transfected with ssRNA of S4 of BTV-9 and infected subsequently with BTV-1 at m.o.i. 3. Based on our previous studies where we demonstrated that only a single replication cycle occurs during 16–18 h of BTV infection, viral cores were isolated from transfected/infected cells after an incubation period of 16 h, allowing only one replication cycle (Matsuo *et al.*, 2010; Matsuo & Roy, 2009). An aliquot of transfected/infected cell lysate was stored as a control sample. Newly synthesized cores were isolated and purified by sucrose gradient centrifugation, and any residual ssRNA potentially not packaged was removed by incubation of the core preparation with RNase A followed by purification with phenol/chloroform extraction and ethanol precipitation. The RNA was then reverse transcribed using primers specific for S4 of BTV-9. To detect the infecting virus and for sample standardization, S6 of BTV-1 was amplified using primers specific for S6 of BTV-1 for every sample. Each DNA product was amplified by PCR, detected by agarose gel electrophoresis and sequenced. In order to distinguish the source of S4, either from virus infection or from transfected mutant S4, two different serotypes of BTV were used: BTV-9 was used to generate mutant S4 and BTV-1 was used for virus infection. Although the S4 sequence was highly conserved among different serotypes, the sequence difference between the two serotypes was sufficient to design differential primers. The 3′ UTR of S4 was composed of 38 nt including the conserved terminal CUUAC, whereas the 5′ UTR consisted of only 3 nt in the non-conserved region in addition to the conserved GUUAA at the 5′ end. A range of S4 mutants with changes in either the 5′ or 3′ UTR, or both, was generated (Fig. 1a, S4.1–S4.7) and tested for their packaging capacity in the *in vivo* packaging assay (Fig. 1b). Full-length S4 of BTV-9 was packaged successfully into BTV-1 cores, demonstrating that S4 of BTV-9 could potentially reassort with BTV-1 (Fig. 1c, upper panel, lane WT). Deletion of the 3′ conserved sequence CUUAC (S4.7) did not affect the packaging of S4, unlike the deletion of the 5′ UTR (conserved sequence GUUAA plus the 3 nt non-conserved AAC sequence) (S4.1), which abrogated packaging of the mutant segment. In addition, upon deletion of the complete 5′ UTR as well as the 3′ UTR, with or without the conserved regions (S4.6 and S4.3, respectively), mutant S4 could no longer be detected in the *in vivo* packaging assay (Fig. 1c, upper panel, lanes S4.3 and S4.6). S6 of BTV-1 was present in all samples, indicating that the particles that did not package mutant S4 of BTV-9 still packaged BTV-1 RNA (Fig. 1c, middle panel). S4 of BTV-9 was detected at a similar level in all control samples of cell lysate followed by transfection/infection, indicating that the transfection of S4 of BTV-9 was successful in all samples (Fig. 1c, lower panel). As deletion of the complete 5′ UTR completely disrupted packaging (S4.1), but deletion of the short non-conserved sequence of the 5′ UTR (S4.5) had no effect on packaging of S4, it was concluded that S4 did not contain segment-specific packaging signals in its 5′ UTR (the GUUAA sequence was strictly conserved among all segments in BTV), but only in its 3′ UTR.

**Fig. 2.**
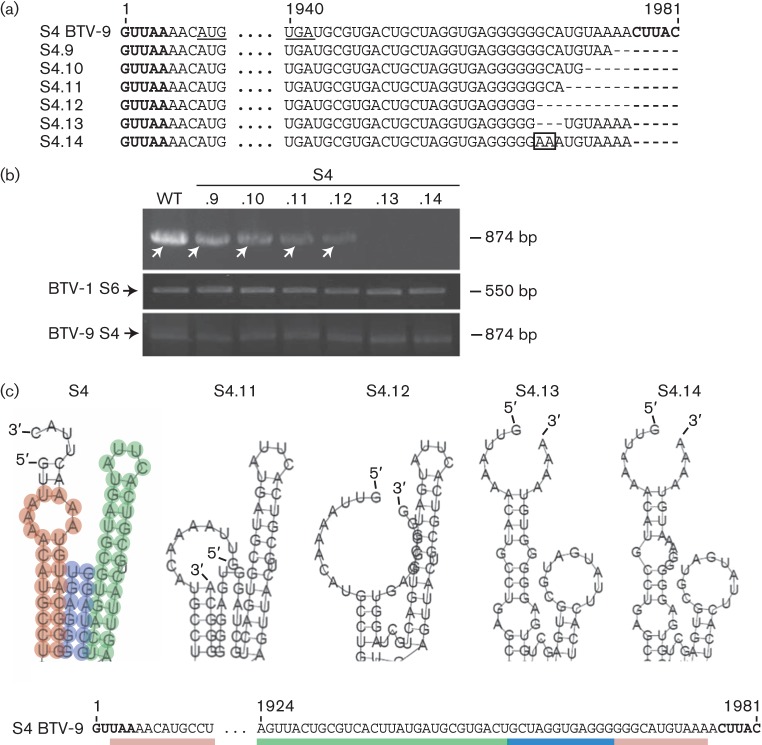
Packaging ability and secondary structure prediction of S4 with mutations in the 3′ UTR. (a) Mutations in the S4 3′ UTR (S4.9–S4.14). The 2 nt substitution in mutant S4.14 is boxed. (b) Upper panel: detection of WT or mutant S4.9–S4.14 ssRNAs in the reverse transcription packaging assay by reverse transcription PCR yielding an 874 bp product (white arrows). Middle and lower panels: as described in Fig. 1. For consistency, each experiment was performed twice. (c) Detail of the secondary ssRNA structures of WT S4 (S4 of BTV-9) and mutants S4.11–S4.14 as predicted using RNAfold prediction software. The hairpin loop of the putative packaging motif for the WT segment (S4) is shown in green, the stem–loop in red and the flexible stretch in blue.

To map further the sequence involved in packaging of S4, the segment was truncated by 7, 10, 12 and 15 nt of the 3′ end of the 3′ UTR, including the conserved terminal 5 nt (Fig. 2a, S4.9–S4.12). Deletions of up to 15 nt of the 3′ UTR (S4.12) did not abrogate the packaging of S4 (Fig. 2b, upper panel, lanes S4.9–S4.12). Although this was a qualitative analysis, S4.12 appeared to be packaged less efficiently. Interestingly, when the conserved CUUAC and an extra 3 nt, GCA (nt 1967–1969), were removed, packaging was abolished completely (S4.13). Furthermore, when GC of these 3 nt were replaced with adenosines, creating AAA (S4.14), S4 was also not detectable (Fig. 2b, upper panel, lanes S4.13 and S4.14). S6 of BTV-1 and S4 of BTV-9 controls were detected in all samples with similar strength (Fig. 2b, middle and lower panels), indicating that the decrease or disappearance of S4 of BTV-9 was not due to poor sample preparation or transfection. These results appeared to be contradictory to the data obtained with S4.12, a mutant that did not contain these 3 nt, but could still be packaged, suggesting that the 3 nt did not influence packaging merely through their sequence, but through a conformational signal yet to be identified.

### Prediction and confirmation of a secondary structure as packaging signal

As the results of the *in vivo* packaging assay pointed to a potential conformational motif being involved in packaging of S4, the secondary structures of WT and S4 mutants were predicted using the prediction software RNAfold. The predicted secondary structure of S4 of BTV-9 suggested an interaction between the 5′ and the 3′ ends of the ssRNA molecule. The two ends formed a structure composed of a hairpin loop (Fig. 2c, shown in green) and a stem–loop (in red) separated by a potentially flexible stretch of nucleotides (in blue). Deletion of the conserved region and the preceding 7 nt (S4.11) did not alter the predicted secondary RNA structure and in fact this mutant S4 could be detected in the *in vivo* packaging assay (Fig. 2b, lane S4.11). Deletion of the conserved region and the preceding 10 nt (S4.12) eliminated the flexible stretch of the predicted secondary RNA structure; however, the longer hairpin was not influenced. This mutant S4 was packaged in the *in vivo* packaging assay. In contrast, the deletion (S4.13) of GCA (nt 1967–1969) or its replacement (nt 1967–1969) with adenosines (S4.14) changed dramatically the predicted secondary structure of the RNA molecule, eliminating the hairpin loop (Fig. 2c), and more importantly, these S4 mutants were not packaged in the *in vivo* packaging assay (Fig. 2b).

**Fig. 3.**
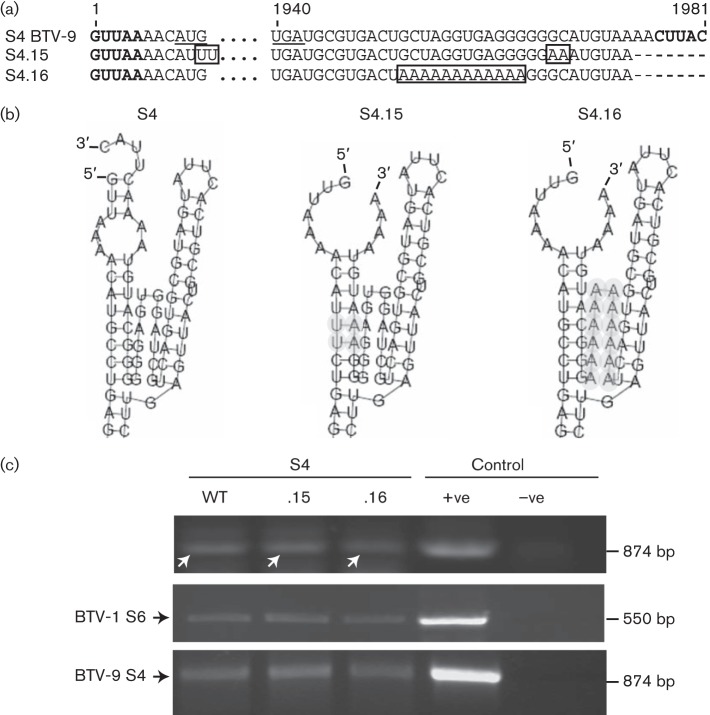
Importance of the structure rather than the sequence for packaging of S4. (a) Nucleotide sequences of ssRNA of mutants S4.15 and S4.16. Boxes indicate mutations. (b) RNAfold secondary RNA structure prediction of S4 mutants. Mutations are depicted in grey. (c) Upper panel: detection of S4 WT or mutant S4.15 and S4.16 RNAs in the reverse transcription packaging assay yielding an 874 bp product (white arrows). Controls for reverse transcription PCR: +ve, S4 of BTV-9 ssRNA; –ve, S4 of BTV-1 ssRNA. Middle and lower panels: as described in Fig. 1. For consistency, each experiment was performed twice.

In order to obtain further evidence that a conformational RNA structure was involved in packaging, we repaired the disruption of the secondary structure caused by the replacement of 2 nt (GC) with adenosine at positions 1967 and 1968 (see S4.14). The structure prediction software was used to identify the nucleotides at the 5′ end of the RNA molecule that were involved in interaction with the essential 3 nt at the 3′ UTR. These nucleotides at the 5′ end were then substituted with uracil (Fig. 3a, S4.15), thus allowing for interaction with the 3 nt at the 3′ UTR replaced previously with adenosines (Fig. 3b, S4.15). This RNA molecule was packaged successfully into viral cores (cores were detected by a control reverse transcription PCR; Fig. 3c, middle panel), confirming the importance of RNA structure rather than sequence in the packaging process of S4 of BTV (Fig. 3c, upper panel, lane S4.15). Similarly, the exchange of the flexible stretch of sequence by adenosines in mutant S4.16 (Fig. 3a, b), which retained the predicted RNA structure intact, still permitted packaging similar to S4.15 (Fig. 3c, upper panel, lane S4.16).

**Fig. 4.**
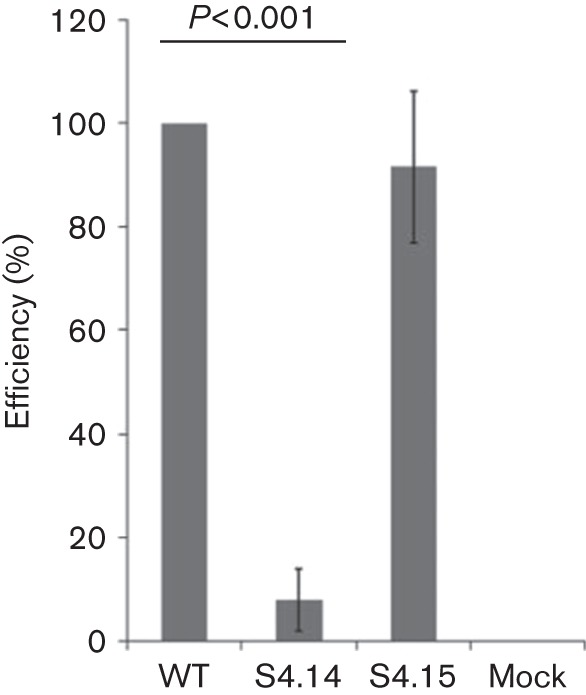
Quantification of the packaging efficiency of mutant segments S4.14 and S4.15 in the reverse transcription packaging assay. Packaged S4 of BTV-9 was quantified by quantitative real-time PCR and correlated with the total amount of BTV cores using S6 as an internal control to determine the packaging efficiency. The efficiency for each mutant was then compared with the efficiency of the WT S4, considered as 100 %. Cells infected with BTV-1 but not transfected with S4 of BTV-9 (mock) were included as control. A difference between S4.14 and S4.15 was confirmed by Student’s *t*-test (*P*<0.001). Error bars: standard deviation from three independent experiments.

To further substantiate the qualitative reverse transcription PCR data and quantify these results, a quantitative real-time PCR was performed to compare packaging efficiency between S4.14 and S4.15 as representatives (Fig. 4). S4.15 had a moderately decreased mean packaging efficiency, although not significant compared with the WT S4 of BTV-9, whilst the packaging efficiency of S4.14 was decreased significantly (*P*<0.001). A mock control showed that the primers used for quantitative real-time PCR were specific for S4 of BTV-9. These results are consistent with the qualitative analysis, confirming that, whilst the S4.14 segment was not detectable by reverse transcription PCR (Fig. 2b), packaging of S4.15 was clearly identifiable (Fig. 3c). Together these results suggest that the conformational motif in S4 regulated packaging of S4 through its RNA secondary structure rather than the sequence itself.

### Confirmation of packaging signal by a cell-free assay system

**Fig. 5.**
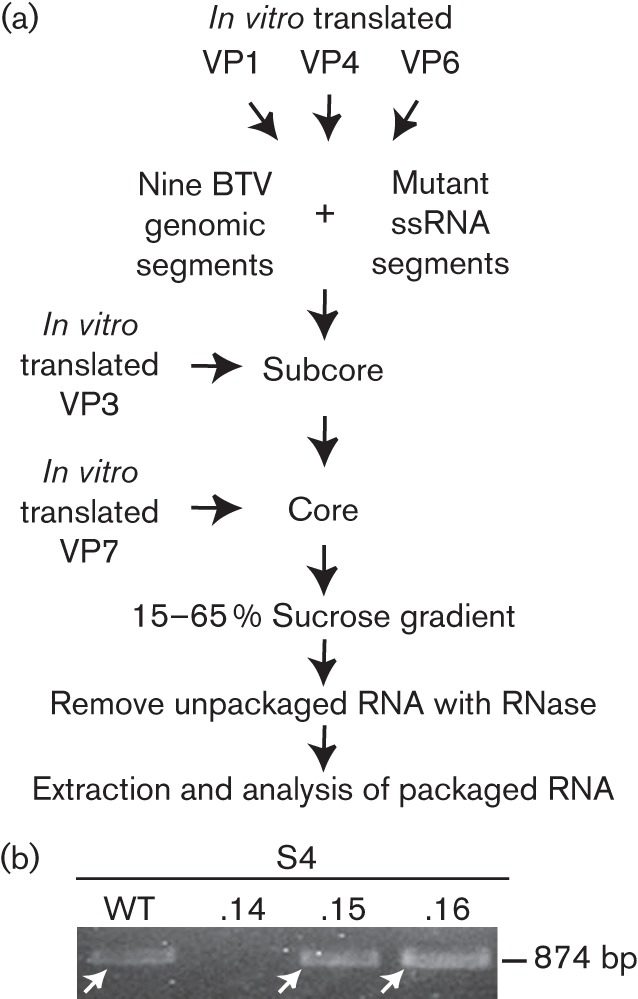
Analysis of packaging competence in the reverse transcription CFA packaging assay. (a) Flow chart of the CFA method. *In vitro* translated VP1, VP4 and VP6 were added to T7 transcripts of 10 BTV genomic segments. Sequentially, VP3 and VP7 were added to the mixture and unincorporated transcript RNase was digested. Cores were purified by sucrose gradient centrifugation and packaged transcripts were purified. (b) WT S4 of BTV-9 or mutant S4.14, S4.15 or S4.16 segments were mixed with T7 transcripts of the remaining nine genomic segments of BTV-1 and used for the CFA assay. Packaged RNA was detected by reverse transcription PCR, yielding a fragment of 874 bp (white arrows) as described for the reverse transcription packaging assay.

Our recently established CFA assay suggests that packaging of BTV segments can occur without mediation of BTV non-structural proteins (Lourenco & Roy, 2011). However, as in infected cells virions are assembled within the cytoplasmic VIBs consisting predominantly of the viral protein NS2, a role for NS proteins in sorting and assembly cannot be ruled out completely. To exclude the possibility that the secondary RNA structure identified was involved in processes other than packaging, some of the S4 mutants were tested in the CFA packaging assay. Viral cores were reconstituted by adding *in vitro* translated BTV proteins sequentially to uncapped T7 transcripts of the nine genomic BTV segments plus mutant S4 (Fig. 5a). Unincorporated ssRNAs were digested with RNase, whilst the packaged RNAs of BTV were protected from digestion by assembled cores. Packaged S4.14, S4.15 and S4.16 were detected either using radiolabelled ssRNAs (data not shown) or by reverse transcription PCR using specific primers (Fig. 5b). Of the three mutants, only S4.14 ssRNA was not packaged. The packaging of the other two mutant ssRNAs into the core was, however, detectable. Although the CFA assay was performed for relevant segments only, results obtained were consistent with the *in vivo* packaging assay and thus substantiated the *in vivo* data (Figs 2b and 3c).

### Mutation in the 3′ UTR does not affect translation

**Fig. 6.**
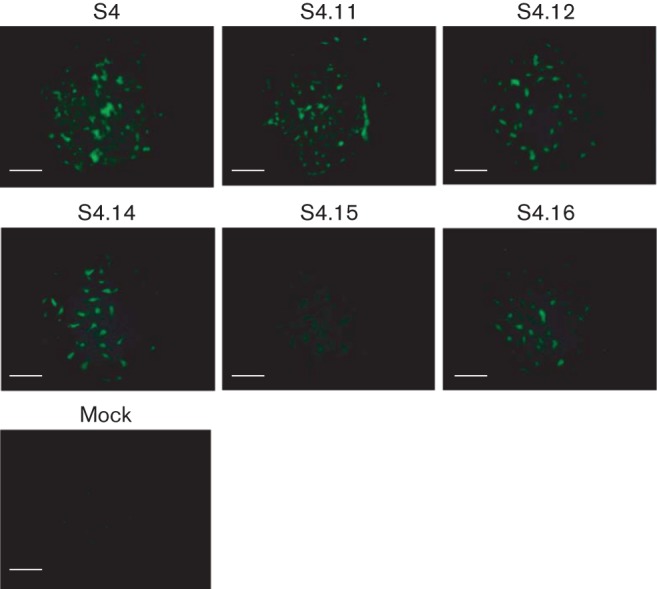
Expression of VP4 from mutant S4 ssRNA. BSR cells were transfected with T7 transcripts of S4 of BTV-9 mutated in the 3′ UTR (S4.11, S4.12, S4.14, S4.15 and S4.16). As controls, WT S4 (B9) and mock-transfected cells were included. After 24 h, cells were fixed and stained with an anti-BTV-9 polyclonal serum. Bars, 200 µm.

It is very likely that the genome of BTV contains several signals governing multiple processes important to viral replication, such as sorting and packaging of genomic segments, formation of dsRNA, protein synthesis regulation or virus morphogenesis. In order to test whether the RNA conformation, identified as being important in packaging of S4, played a role in VP4 protein translation, each S4 mutant ssRNA transcript was generated *in vitro* from T7 constructs for five different S4 mutants (Figs 2 and 3): S4.11 (12 nt), S4.12 (15 nt), S4.14 (5+2 nt substitution), S4.15 (same as S4.14 plus 2 nt substitutions that eliminate the ORF ATG) and S4.16 (adenosine stretch). BSR cells transfected with ssRNAs were incubated for 24 h, then stained with an antiserum raised against BTV-9, and VP4 expression was visualized (Fig. 6). Although fluorescence signals were not strong due to the nature of ssRNA transfection, substitutions or deletions of either parts or all of the 3′ UTR of S4 did not seem to influence the expression of VP4 and confirmed the transfection efficiency of T7-derived S4 transcripts. As expected, the only mutant which did not express VP4 was S4.15, where the G of the start codon of the ORF was exchanged for U in order to reconstitute the predicted secondary RNA structure. Therefore, it seems as if the 3′ UTR did not contain any signal necessary in initiation of translation mediated by cellular components. However, it was shown recently that the conserved 3′ end of BTV segments consisting of the sequence CUUAC is essential and sufficient for upregulation of translation of BTV proteins by viral NS1 (Boyce *et al.*, 2012).

### Prediction of sequence requirements for packaging of S4 containing ORF of S8 of BTV-9

**Fig. 7.**
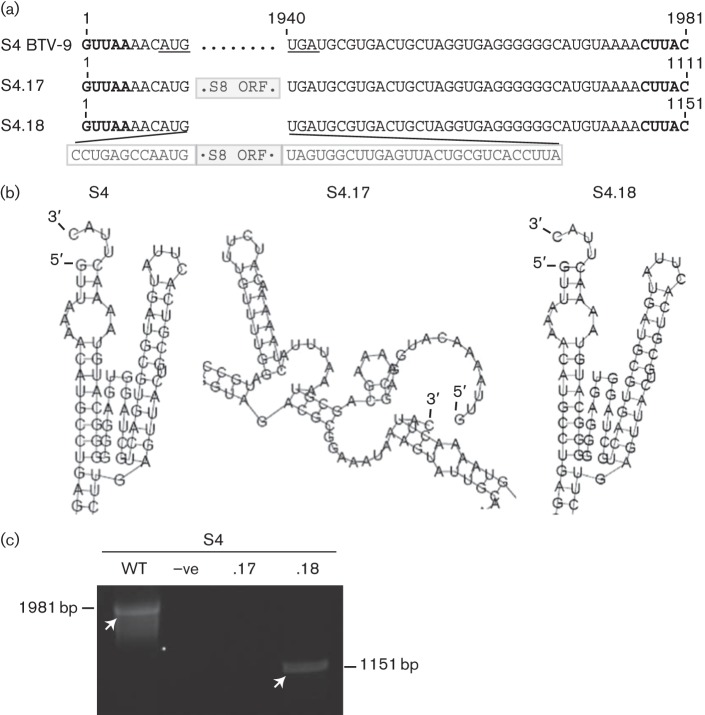
Prediction and confirmation of minimal sequence requirements for packaging of chimeric S4 segments. (a) Nucleotide sequence of chimeric segments. Boxed sequence represents S8 of BTV-9 ORF (grey box, S4.17 and S4.18) or flanking sequences from S4 of BTV-9 ORF (white box, S4.18). (b) RNAfold structure prediction of S4.17 and S4.18. (c) Reverse transcription PCR for S4 of BTV-9 (WT) without S4 (–ve), S4.17 or S4.18 from the CFA assay. Arrows indicate bands of 1981 and 1210 bp for mutant and WT ssRNAs, respectively. For consistency, each experiment was performed three times.

To further support the importance of the RNA structural motif required for packaging, we attempted to predict the minimum S4-specific sequence within the 5′ and 3′ ends required to sort and package an S4 that had its ORF replaced by the ORF of S8 of BTV-9 (Fig. 7a). Using the RNA structure prediction program RNAfold, we found that replacing the ORF of S4 with the ORF of S8 (S4.17) disrupted the putative packaging motif (Fig. 7b). However, addition of the first 20 nt of the 5′ end and the last 65 nt of the 3′ end of S4 (S4.18) was sufficient to adopt the RNA motif structure predicted to be necessary for packaging when the RNA molecule contained the ORF of S8 (Fig. 7b). The two chimeric segments (S4.17 and S4.18) were generated and tested both in the *in vivo* packaging assay (data not shown) and in the CFA packaging assay (Fig. 7c). In both assays, S4.18 was packaged successfully, whereas S4.17 was not, confirming that the structural motif was indeed the mediator of S4 packaging.

### Hairpin loop secondary structure is also important for BTV S8 packaging

**Fig. 8.**
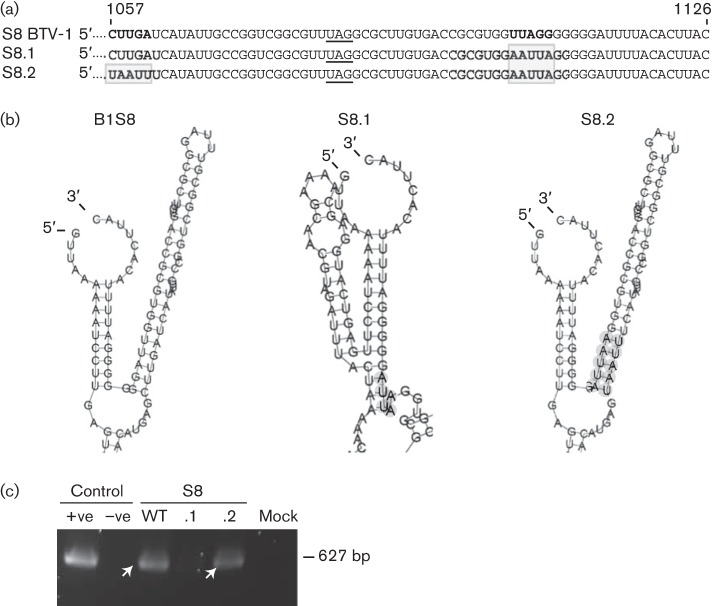
Relevance of the RNA secondary structure for packaging of S8 of BTV-1 analysed by the CFA assay. (a) S8 of BTV-1 3′ terminal sequence (69 nt). Nucleotides predicted to be important for the secondary structure are indicated in bold and the mutated nucleotides are boxed. The STOP codon is underlined. (b) Predicted RNA structures of mutant (S8.1 and S8.2) or WT (B1S8) ssRNA segments. The region targeted in each mutant segment is indicated in grey. (c) Packaging was analysed by reverse transcription PCR (fragment of 627 bp indicated by white arrows) of mutant (S8.1 and S8.2) and WT ssRNA. S8 of BTV-1 (+ve) or no ssRNA (–ve) as positive or negative reverse transcription PCR controls. Mock, CFA excluding S8.

We demonstrated that the hairpin loop structure was essential for packaging of S4 of BTV-9, whilst it was unclear whether this structure was specific for this segment of BTV-9 or the function of this structure could also apply to other segments of other serotypes. To investigate if a similar secondary structure in other BTV ssRNA segments was involved in packaging, we undertook the packaging assay with an alternative segment of a different serotype, S8 of BTV-1. S8 encodes the non-structural protein NS2. Similar to S4 of BTV-9, S8 of BTV-1 also has a long hairpin structure next to the stem–loop structure according to RNA structure prediction (Fig. 8b, B1S8). Based on this computational analysis, 5 nt, UUAGG, located at the base of the hairpin structure (nt 1104–1108) were substituted by AAUUA (S8.1). This substitution mutation was predicted to destroy the hairpin loop as the nt 1104–1108 region could no longer complement the corresponding sequences CUUGA (nt 1057–1061; Fig. 8a, b). This mutant segment was then tested in the CFA packaging assay, which demonstrated that it could no longer be packaged into viral cores (Fig. 8c, lane S8.1). In parallel, a compensatory mutation was also designed to change the CUUGA to UAAUU, which would recover the secondary hairpin loop structure (Fig. 8a, b, S8.2). This mutant was packaged successfully in the *in vitro* CFA assay (Fig. 8c, lane S8.2). These results suggested that the hairpin loop structure might serve as a conformational packaging signal for other ssRNA segments of different BTV serotypes.

## Discussion

How a segmented virus packages a full set of genomic segments in a single particle is still poorly understood. A number of studies on other segmented viruses such as phi6 (Mindich, 2004) or influenza virus (Chou *et al.*, 2012; Goto *et al.*, 2013) suggest that it is a highly selective and controlled process. In contrast to these viruses, information on the RNA packaging signals for the members of *Reoviridae* is less understood. Recent reports on rotavirus sequences suggest some conserved *cis*-acting structural elements within the genomic segments, implicating their importance in rotavirus biology (Biswas *et al.*, 2014; Desselberger *et al.*, 2013; Li *et al.*, 2010). To the best of our knowledge, this is the first report on the nature of the packaging signal for two BTV segments analysed using *in vivo* and *in vitro* systems. S4 of BTV-9 was shown to code for a putative structural motif in its ssRNA molecule which is responsible for its packaging. The predicted structure of S4 ssRNA comprises a stem–loop and a hairpin loop connected by a flexible stretch formed by interaction of the 3′ and 5′ ends of the RNA molecule. For the first time to our knowledge, an assay was applied which allowed for the investigation of packaging signals independently of other signals potentially important in the biology of the virus. Investigation of packaging signals of viruses with segmented genomes has so far relied on *in vivo* assays, such as reverse genetics, which allows introduction of mutations in the viral genome directly or other infection-based test systems. In these assays, no distinction can be made between signals which govern sorting, packaging, translation, transcription or other processes involved in viral replication. A recently established assembly assay for BTV allowed the study of packaging processes independently of a cellular system (Lourenco & Roy, 2011), thus eliminating possible influences of other processes important in viral replication.

In a previous report, the regions essential for viral replication on S9 of BTV-1 were mapped (Matsuo & Roy, 2009). The 3′ UTR was shown to be essential for replication as well as sequences of the 5′ and 3′ ends of S9 extending into the VP6 ORF of S9 when the ORF of enhanced green fluorescent protein (EGFP) was inserted. These results are consistent with those obtained in this current study as the 3′ UTR was shown to be part of a structured RNA motif important in packaging of S4. Formation of particular RNA structures depends strongly on the entire RNA sequence as the RNA molecule adopts a structure corresponding to the state of lowest energy. In order to tolerate a BTV non-specific sequence such as EGFP within a particular BTV segment, the 3′ and 5′ ends of the segment must contain sufficient segment-specific sequence to ensure the structural motif for packaging is still formed. In this current study it was predicted and shown that the exchange of the S4 ORF (VP4) with the S8 ORF (NS2) of BTV-9 could guarantee packaging of this chimeric segment only if 20 nt of the 5′ end and 65 nt of the 3′ end of S4 were still present. However, other elements may also be involved in the formation and stabilization of the structures important for packaging.

*Cis*-acting functional RNA elements have long been proposed to be involved in genome sorting and packaging of viruses with segmented RNA genomes. Similar to BTV, ssRNA of *Rotavirus*, another member of the family *Reoviridae*, is predicted to contain sequences in its 5′ and 3′ UTRs which form a panhandle structure with stable stem–loops within (Chen & Patton, 1998; Li *et al.*, 2010; Tortorici *et al.*, 2006). These stem–loops differ between the rotavirus RNA segments and are suggested to be important in assortment of segments. Likewise, the termini of influenza virus RNA form a panhandle-shaped motif with stem–loop structures predicted to reside adjacent to the motif and suggested to mediate packaging (Hutchinson *et al.*, 2010). Panhandle plus stem–loop structures as motifs for genome segment sorting and packaging might be a common feature in segmented RNA viruses. To the best of our knowledge, however, our study is the first to present experimental evidence for a structural motif being involved in segment packaging. Moreover, we also demonstrated that a similar structural motif exists for a different RNA segment of another serotype (S8 of BTV-1) and that it is functionally important.

The CFA packaging assay employed in this work relies on the interaction of structural proteins with the viral RNA. Participation of cellular factors is unlikely as a plant-based extract was used for *in vitro* translation, but cannot be ruled out entirely. The *in vivo* packaging assay is based on assembly of viral cores within infected cells transfected previously with a mutated segment. The modified segment competes directly for sorting and packaging with the WT segment in the presence of all viral proteins. NS2 is known to interact with BTV ssRNA and is therefore believed to play a role in RNA packaging specifically (Lymperopoulos *et al.*, 2006; Markotter *et al.*, 2004). Additionally, NS1 was shown to be a major regulator of viral protein synthesis, which could influence the efficiency with which viral RNA can be packaged (Boyce *et al.*, 2012). The exact role of both proteins in BTV morphogenesis is not yet clear, but the fact that mutant S4.14 could not be detected in the CFA assay (Fig. 5b), but was weakly packaged in the *in vivo* packaging assay (8 %, Fig. 4), suggests an important role of other cellular and viral proteins during assembly. Further quantitative investigation of displacement of WT segments by modified segments using the *in vivo* and CFA packaging assay can help to gain more insight into the sorting of viral segments.

Note that similar putative loop structures in other BTV strains, such as BTV-1, BTV-4, BTV-7, BTV-16 and BTV-20, could also be identified using the same or slightly adjusted prediction parameters (data not shown), implying that S4 of many BTV serotypes probably contains equivalent loop structures. Similar secondary structures could also be predicted at lower temperatures for S4 of BTV-9 by using an alternate RNAfold prediction program. It is worth noting that the actual RNA structure can only be studied using new techniques such as SHAPE-Seq (selective 2′-hydroxyl acylation analysed by primer extension and sequencing), which will be followed up in our future studies.

However, it cannot be excluded that sorting and packaging signals are serotype specific. In fact, it has been shown in cell culture experiments as well as in natural infection that BTV segments do not reassort at a rate expected by statistical calculations when 10 genomic segments are involved (Ramig *et al.*, 1989; Samal *et al.*, 1987). Certain segments of specific serotypes seem to reassort more easily than others. One reason for this phenomenon might be different sorting and packaging signals between serotypes, although other factors such as virus fitness should also be considered. In addition, it cannot be excluded that the sequences which form the sorting and packaging signals are employed in other processes of virus replication, such as regulation of protein translation or initiation of dsRNA synthesis.

The findings in this study will be of great use in investigating BTV biology. Prediction of the sequence requirement of a chimeric segment, to be successfully sorted and packaged, will allow the rational design of modified BTV, which can carry any sequence as long as other limiting factors such as length or toxicity of the protein product of the chimeric segment are considered. Once the structural motif for packaging is identified, this knowledge can be used to create segments carrying heterologous sequences which will be sorted and packaged into virion particles. This includes construction of viruses carrying marker genes, which will allow live-cell imaging in stable or inducible complementing cell lines for the deleted BTV protein. In vaccine development, multivalent disabled infectious single-cycle vaccines could be designed rationally, carrying coding sequences of immunogenic VP2 or VP5 proteins of different serotypes. The test systems used in this study can be applied to other BTV segments or other segmented RNA viruses.

## Methods

### 

#### Plasmids, DNA templates and virus strains.

To generate T7 transcripts for use in the CFA packaging assay and the reverse genetics system, cDNA of exact copies of each BTV-1 (GenBank accession numbers FJ969719–FJ969728) genome segment and cDNA of S4 (KF769399.1) and S8 of BTV-9 were derived from viral dsRNA using the method of full-length amplification of cDNA as described previously (Boyce *et al.*, 2008).

#### Generation of WT and mutant T7 transcripts.

Mutants were generated using different 3′ and 5′ primers encoding T7 promoter (available upon request). The capped and uncapped ssRNAs were generated as described previously (Boyce *et al.*, 2008; Lourenco & Roy, 2011).

#### Prediction of RNA secondary structures.

RNAfold from the Vienna RNA Webserver (http://rna.tbi.univie.ac.at/cgi-bin/RNAfold.cgi) was used for this study. The parameters used for RNA predictions were the standard for this software when this work was initiated. The energy parameters used were Turner model 1999; fold algorithms: minimum free energy; dangling options: dangling energies on both sides of a helix in any case. Temperature set: 37 °C.

#### *In vivo* packaging assay.

BSR cells were transfected with 5 µg *in vitro* generated uncapped T7 transcripts of S4 of BTV-9 ssRNAs using Lipofectamine 2000 as described previously (Boyce *et al.*, 2008). The cells were infected subsequently with BTV-1 (m.o.i. 3). After incubation for 16 h allowing for one replication cycle to be completed, viral cores were prepared as described previously (Boyce *et al.*, 2008). Briefly, cells were lysed and the clarified supernatant was loaded onto a 40 % (w/v) sucrose cushion. After ultracentrifugation at 141 000 ***g*** for 2 h at 20 °C, the pellet containing viral cores was resuspended in RNase-free water. RNAs associated with cores but not encapsidated were digested with RNase A (1 µg µl^−1^) for 1 h at 35 °C. Genomic RNA was then extracted, precipitated and subjected to reverse transcription PCR or quantitative real-time PCR with primers specific for internal (non-UTR) sequences of BTV-9. The same conditions of transfection were used for each experiment and transfection of each group was performed at the same time for each individual experiment. Individual experiments were performed three times to confirm the consistency of results.

#### Reverse transcription PCR.

S4 of BTV-9 from the *in vivo* packaging assay was detected by reverse transcription PCR using ReverseAid Premium Reverse Transcriptase (Thermo) and BioMix Red PCR master mix (Bioline) following the standard protocols suggested by the manufacturers. The following primers were used: for S4 mutants, S4-BTV9F 5′-TTATTTACCTATATGGCATC-3′ and S4-BTV9R 5′-CTTGGCTGGTATCCTATG-3′; for detection of S4.17 and S4.18, S8-BTV9F 5′-GAGCCGCTCCTAGCGCAAATC-3′, and for the S4 3′ end, 5′-GTAAGTTTTACATGCCCCCCTCACCTA-3′; for detection of S6 of BTV-1 as control B1S6 qF 5′-AAAAAGTTCTCTAGTTG-3′ and BTV-1 M6 554R 5′-GGATCGATCCAATCATCAGCCGCATC-3′.

#### Quantitative real-time PCR.

S4 of BTV-9 was quantified by the 7500 Fast Real-Time PCR system and SYBR Select Master Mix (Applied Biosystems), using primers B9S4F 5′-TGGCATCTGACAGGAAACGAAAGCT-3′ and B9S4R 5′-TATACAGTGATATTCATCCCTCTTGGC-3′. The copy number was then correlated with the total BTV cores in the same sample. As an internal control for total BTV RNA copy numbers, S6 was measured as standard using specific primers as reported by Toussaint *et al.* (2007).

#### Cell-free *in vitro* packaging assay.

The CFA packaging assay was carried out as described previously (Lourenco & Roy, 2011). Briefly, VP1, VP4, VP6, VP3 and VP7 were *in vitro* translated sequentially using wheatgerm extract (Promega) and mixed with the 10 BTV uncapped ssRNAs to form viral cores. The whole mixture was then fractionated through a continuous sucrose gradient and tested for RNA content. In relevant fractions, unpackaged RNAs were eliminated by RNase One (Promega) digestion. Packaged ssRNAs were detected by reverse transcription PCR using primers for S4 of BTV-9 as described above (for Fig. 5) or S4 of BTV-9 5′ and 3′ end primers: SBF1-T7-S4-BTV9, 5′-AAAACCTGCAGGTAATACGACTCACTATAGTTAAAACATGCCTGAGCCAC-3′; S4-BTV9-terminus, 5′-GTAAGTTTTACATGCCCCCC-3′ (for Fig. 7). Primers for S8 of BTV-1 were: for reverse transcription reaction of S8, B1S8R 5′-GTAAGTGTAAAATCCCCC-3′; for detection of S8, B1S8F 5′-GTTAAAAAATCCTTGAGTCATGGAG-3′; B1S8 627R, 5′-CAGCTTCTCCAATCTGCTGG-3′.

#### Immunofluorescence staining.

BSR cells (2×10^5^ per well) were seeded on coverslips on a 24-well-plate (Nunc). After 24 h incubation cells were transfected with 1 µg mutant S4 ssRNAs using Lipofectamine 2000 (Invitrogen) according to the manufacturer’s instructions. Cells were incubated for 24 h at 37 °C, fixed with 4 % paraformaldehyde for 30 min at room temperature and permeabilized subsequently with 0.1 % Triton X-100 in PBS. Staining was performed with a BTV-9-specific sheep antiserum followed by incubation with FITC-labelled anti-goat/sheep IgG antibody (Sigma). Images were taken with a Nikon Eclipse TS100 microscope and a Nikon Coolpix 990 camera.
